# Coinfection of *Porphyromonas gingivalis* and *Toxoplasma gondii* impairs neurocognitive function and induces anxiety-like behavior in rats: a behavioral study

**DOI:** 10.3389/fimmu.2026.1728337

**Published:** 2026-01-30

**Authors:** Henglong Cao, Jianfeng Lin, Wanyi Wei, Jianzhao Luo, Hao Yuan, Yining Song, Kunmei Yang, Xiao Ma, Ning Song, Miao Yu

**Affiliations:** 1 School and Hospital of Stomatology, Guangdong Engineering Research Center of Oral Restoration and Reconstruction, Guangzhou Key Laboratory of Basic and Applied Research of Oral Regenerative Medicine, Guangzhou Medical University, Guangzhou, China; 2 Guangdong Provincial Key Laboratory of Zoonosis Prevention and Control, College of Veterinary Medicine, South China Agricultural University, Guangzhou, Guangdong, China; 3 Guangdong Academy of Medical Sciences, Guangdong Provincial People’s Hospital, Guangzhou, Guangdong, China; 4 Department of stomatology, Shenzhen Children’s Hospital, Shenzhen, Guangdong, China; 5 Department of Oral Health Sciences-BIOMAT, KU Leuven and Dentistry, University Hospitals Leuven, Leuven, Belgium

**Keywords:** anxiety, cognitive function, *P. gingivalis*, periodontitis, *T. gondii*

## Abstract

*Toxoplasma gondii* (*T. gondii*) is a ubiquitous protozoan parasite that can infect a wide range of hosts, including humans and rodents. Dental plaque microbiota serves as the initiating factor in periodontal diseases, with *Porphyromonas gingivalis* (*P. gingivalis*) being the principal pathogenic bacterium. Recent studies have suggested a potential link between *T. gondii* infection, periodontal diseases and neuropsychiatric disorders, although the underlying mechanisms remain unclear. This study aimed to investigate the effects of *T. gondii* infection on cognitive function and anxiety-like behavior in rats with periodontitis, using a series of behavioral tests, including the Morris water maze, open field test, novel object recognition test, Y-maze test, and elevated plus maze test. We explored whether coinfection of *P. gingivalis* and *T. gondii* could impair spatial learning and memory and induce anxiety-like behavior in rats. Our results showed that coinfection of *P. gingivalis* and *T. gondii* significantly increased anxiety-like behavior and reduced cognitive function in rats. These findings suggest that the coinfection may disrupt central nervous system (CNS) function, providing new insights into the association between *T. gondii* infection, periodontitis, and neuropsychiatric comorbidities. Future research should focus on elucidating the molecular mechanisms underlying these effects and exploring potential therapeutic strategies to mitigate the impact of *T. gondii* infection and periodontal diseases on mental health.

## Graphical Abstract

**Figure:**
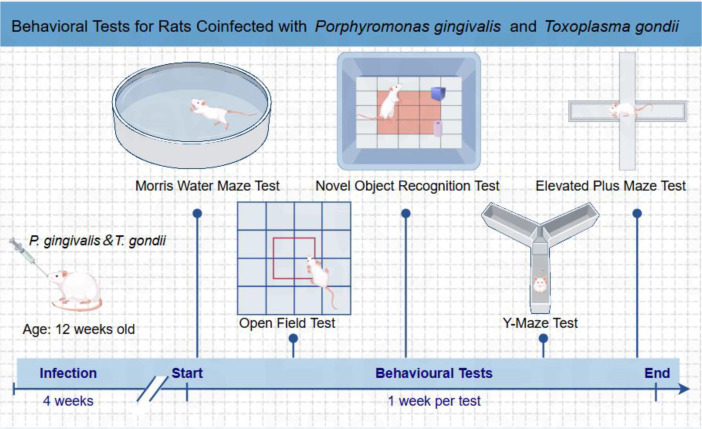

## Introduction

Neurocognitive functions are critical mental processes that include memory, attention, executive function, and emotional regulation. Common factors that can influence these functions are infections, inflammation, and neurotransmitter imbalances (1). Among these, infections with certain pathogens, such as *Toxoplasma gondii* (*T. gondii*), have been shown to impact cognitive and behavioral outcomes. *T. gondii* is a ubiquitous protozoan parasite that infects a wide range of warm-blooded animals, including humans and rodents (1, 2). It is estimated that about one-third of the global human population is chronically infected with *T. gondii*, making it one of the most successful parasitic pathogens (3, 4). While the majority of infections are asymptomatic, *T. gondii* can cause severe disease in immunocompromised individuals and congenitally infected neonates (5). In addition to its well-documented effects on physical health, accumulating evidence suggests that *T. gondii* infection may also influence cognitive and behavioral processes in the host (6, 7). Over the past few decades, numerous studies have reported a potential association between *T. gondii* infection and various neuropsychiatric disorders, including schizophrenia, bipolar disorder, and anxiety (6). Analyses have shown that individuals with schizophrenia are more likely to have antibodies against *T. gondii* compared to healthy controls (8). Similarly, studies have found a higher prevalence of *T. gondii* infection in patients with bipolar disorder and major depressive disorder (9). While the exact mechanisms underlying these associations remain unclear, several hypotheses have been proposed. One of the leading hypotheses is that *T. gondii* infection may alter neurotransmitter levels in the brain, particularly dopamine and glutamate, which are important for normal cognitive and behavioral function (10, 11). *T. gondii* has been shown to change dopamine levels in infected mice, which could contribute to the behavioral changes observed in these animals (12). Additionally, the parasite may induce neuroinflammation through the activation of microglia and the production of pro-inflammatory cytokines, leading to synaptic dysfunction and cognitive deficits (13, 14).

The oral cavity is a complex ecosystem harboring a diverse microbiota that plays a crucial role in maintaining oral health and influencing systemic health (15). The oral microbiome has been implicated in various systemic diseases, including cardiovascular disease, diabetes, and neurodegenerative disorders (15–17). The concept of the “oral-brain axis” has emerged to describe the bidirectional communication between the oral microbiome and the central nervous system (CNS) (17). The oral-brain axis involves several pathways through which oral pathogens and their virulence factors can influence brain function. One key mechanism is the induction of systemic inflammation, which can disrupt the blood-brain barrier (BBB) and lead to neuroinflammation. Oral pathogens such as *Porphyromonas gingivalis* (*P. gingivalis*), a major etiological agent of periodontitis, produce lipopolysaccharides that can trigger the release of pro-inflammatory cytokines (IL-1β, IL-6, TNF-α) and chemokines (CCL2, CXCL8). These inflammatory mediators can enter the bloodstream and cross the BBB, contributing to neuroinflammation and cognitive decline (18). For instance, *P. gingivalis* and its virulence factors have been detected in the brains of Alzheimer’s disease patients, suggesting a direct role for oral pathogens in neurodegenerative processes (19). Additionally, oral pathogens can modulate the host’s immune response, leading to an imbalance in the oral microbiome and exacerbating periodontitis. This, in turn, can further contribute to systemic inflammation and cognitive decline through the oral-brain axis (18).

Periodontitis, a highly prevalent chronic inflammatory disease affecting up to 50% to 80% of the global population, is characterized by the destruction of tooth-supporting tissues, including the gingiva, periodontal ligament, and alveolar bone (19). This widespread condition not only leads to severe oral health issues such as tooth loss but also has significant systemic implications. Periodontitis has been linked to various systemic diseases, including cardiovascular disease, diabetes, and neurodegenerative disorders, highlighting its substantial impact on overall health (19). It is caused by a dysbiotic oral microbiome, with *P. gingivalis* being a key pathogen (16). Recent studies have highlighted a potential link between periodontitis and cognitive decline, particularly in the context of Alzheimer’s disease (17, 20). The mechanisms by which periodontitis may contribute to cognitive decline include the induction of systemic inflammation, the disruption of the BBB, and the direct invasion of oral pathogens into the CNS (17). Additionally, periodontitis has been shown to exacerbate the effects of other risk factors for cognitive decline, such as aging and genetic predisposition (21).

Our previous research has made a hypothesis that *T. gondii* infection may lead to the production of pro-inflammatory cytokine, which may cross the BBB and contribute to neuroinflammation. Additionally, *T. gondii* can modulate the host’s immune response, exacerbating periodontitis. This, in turn, can further contribute to systemic inflammation and cognitive decline through the oral-brain axis (22).

In the present study, we explored whether coinfection with of *P. gingivalis* and *T. gondii* could impair cognitive function and induce anxiety-like behavior in rats. We conducted a series of behavioral experiments in rats infected with *T. gondii*, including the Morris water maze, open field test, novel object recognition test, Y-maze test, and elevated plus maze test. The primary objectives of this study were to assess the impact of *T. gondii* infection and periodontitis on cognitive function and anxiety-like behavior in rats and investigate the association between *T. gondii* infection, periodontitis, and neuropsychiatric disorders.

By elucidating the role of *T. gondii* infection and periodontitis in the neuropsychiatric disorders, we aimed to provide new insights into the pathophysiology of neuropsychiatric disorders and identify potential therapeutic targets for mitigating the cognitive and behavioral consequences of parasitic infections and periodontal diseases.

## Materials and methods

### Experimental animals

A total of 24 male Sprague-Dawley rats, weighing 200–250 g, were obtained from the Laboratory Animal Center of South China Agricultural University. The rats were housed in a controlled environment with a 12-hour light/dark cycle and free access to food and water. The animals were acclimatized to the laboratory conditions for at least one week before the start of the experiments. All experimental procedures were approved by the Institutional Animal Care and Use Committee of South China Agricultural University with registration number 2023f089 and were conducted in accordance with the National Institutes of Health guidelines for the care and use of laboratory animals.

### P. gingivalis culture

The standard strain of *P. gingivalis* ATCC33277 was provided by the Laboratory of Stomatology School, Guangzhou Medical University. The primary culture of *P. gingivalis* was inoculated on solid blood agar plates containing brain heart infusion medium and cultured under anaerobic conditions (85% N_2_, 10% CO_2_, 5% H_2_) at 37°C for 5 to 7 days. Black colonies were observed on the plates. After purification, the strain was subcultured 2 to 3 times in an anaerobic environment. A single colony was then transferred to 5 mL of brain heart infusion liquid medium and cultured anaerobically for 48 hours. The bacterial suspension was centrifuged at 4°C, 3000 rpm for 10 minutes, washed twice with sterile PBS, and resuspended in 1 mL PBS. The bacterial concentration was determined using a UV spectrophotometer at 600 nm, and the suspension was diluted with sterile PBS to a final concentration of 10^9^ CFU/mL (23).

### T. gondii culture

The *T. gondii* Me49 strain used in the experiment was maintained and provided by the Laboratory of the College of Veterinary Medicine, South China Agricultural University. *T. gondii* tachyzoites, stored in liquid nitrogen, were initially reconstituted by intraperitoneal inoculation into Kunming mice with approximately 10^6^ tachyzoites. Mice typically exhibited symptoms such as lethargy, reduced activity, ruffled fur, abdominal distension, tremors, or rapid breathing around day 7, indicating the onset of disease. Examination of the peritoneal fluid revealed a high concentration of *T. gondii*. The peritoneal fluid containing *T. gondii* was then used to passage the infection through mice for three consecutive generations to establish a stable *T. gondii*-infected mouse colony for subsequent experiments (24, 25).

### Induction of periodontitis and *T. gondii* infection model

#### Periodontitis rat model construction

Rats were first lightly anesthetized with 2% isoflurane, followed by intraperitoneal injection of 1 mL of 1.5% sodium pentobarbital solution. Once the rats’ breathing rate decreased and muscles fully relaxed, they were secured on an operating table in a supine position. Their limbs and incisors were fixed, and their mouths were fully exposed using hemostatic forceps. A 3–0 silk suture was wrapped around the cervical areas between the first and second maxillary molars on both sides, and excess suture was trimmed after knotting. The rats’ breathing and heart rate were monitored throughout the procedure (26).

*P. gingivalis* bacterial suspension was centrifuged at 3000 rpm for 5 minutes at 4°C, then resuspended in PBS containing 4% sodium carboxymethyl cellulose, with the concentration adjusted to 10^9^ CFU/mL. Using a syringe, 1 mL of the *P. gingivalis* suspension (10^9^ CFU/mL) was inoculated into the gingival crevices and above the suture lines of the rats’ teeth. This inoculation was repeated every 3 days for a total of 7 times. The rats were fed a standard diet, and the sutures were checked every 3 days. If any suture came loose, it was retied to ensure the successful establishment of the experimental periodontitis model. Periodontitis was verified by three complementary methods: (i) observation of gingival redness, swelling, and bleeding on probing; (ii) micro-CT measurement of alveolar bone loss (cemento-enamel junction to alveolar crest distance) in the distal root of the left maxillary first molar; and (iii) histological examination (H&E staining) of periodontal tissues to confirm inflammatory cell infiltration and crestal alveolar bone (26).

#### *T. gondii* rat model construction

Kunming mice chronically infected with *T. gondii* Me49 strain were anesthetized and euthanized by cervical dislocation. Their brains were removed, homogenized in a mortar, and diluted with saline. A 10 μL aliquot was placed on a slide, and the number of *T. gondii* brain cysts was counted under a light microscope. Rats to be infected with *T. gondii* were then orally gavaged with 1 mL of the homogenate containing 100 brain cysts/mL. Oral gavage of 100 brain cysts was chosen to mimic this natural enteric route and reliably establish chronic CNS infection. Confirmation of infection status was performed by light microscopic counting of *T. gondii* brain cysts in homogenized brain tissue to confirm *T. gondii* infection (27).

#### *P. gingivalis* and *T. gondii* co-infection rat model construction

The same procedures as described above were followed for ligation, *P. gingivalis* inoculation, and *T. gondii* gavage to establish the co-infection model.

### Experimental groups

The rats were divided into four experimental groups (n = 6): (1) Control group (C): The rats received PBS injection and no ligature placement or *P. gingivalis* application. (2) *T. gondii* group (T): The rats were orally gavaged with 100 *T. gondii* cysts. (3) Periodontitis group (P): The rats received ligature placement and *P. gingivalis* application. (4) Coinfection group (P+T): The rats received both ligature placement, *P. gingivalis* application and *T. gondii* infection.

### Behavioral tests

#### Morris water maze test

The Morris water maze test was conducted to evaluate spatial learning and memory in rats (28). The maze consisted of a circular pool (diameter 1.6 m, height 0.5 m) filled with water at 22-24 °C. A hidden platform (10 cm in diameter) was submerged 1 cm below the water surface in one of the four quadrants. The test was performed over 6 days, with 5 days of training trials followed by a probe trial on day 6.

Training Trials: Each rat was given four trials per day for 5 consecutive days. The starting position was varied randomly for each trial. If the rat failed to find the platform within 90 seconds, it was gently guided to the platform and allowed to remain there for 30 seconds. The escape latency (time to reach the platform) and path length were recorded using a video tracking system.

Probe Trial: On day 6, the platform was removed, and each rat was allowed to swim freely for 90 seconds. The time spent in the target quadrant and the number of crossings over the former platform location were recorded.

#### Open field test

The open field test was used to assess locomotor activity, exploratory behavior, and anxiety-like behavior in rats (29). The test was conducted in a square arena (100 cm × 100 cm × 40 cm) with the floor divided into 25 equal squares. The arena was placed in a quiet room with dim lighting. Each rat was placed in the center of the arena and allowed to explore for 5 minutes. The total distance traveled, the number of line crossings, the time spent in the center zone, and the number of rearing events were recorded using a video tracking system.

#### Novel object recognition test

The novel object recognition test was conducted to evaluate cognitive function and memory in rats (28). The test consisted of two phases: training and testing. In the training phase, each rat was placed in a test arena (50 cm × 50 cm × 40 cm) containing two identical objects (A and B) for 5 minutes. After a 1-hour retention interval, the rat was returned to the arena, where one of the familiar objects (B) was replaced with a novel object (B2). The rat was allowed to explore the objects for another 5 minutes. The time spent exploring each object was recorded, and the recognition index (RI) was calculated as follows: RI= Time spent exploring the novel object/Total time spent exploring both objects×100%.

#### Y-maze test

The Y-maze test was used to assess spatial working memory in rats (30). The maze consisted of three identical arms (30 cm long, 10 cm wide, 15 cm high) arranged at 120° angles. Each rat was placed at the end of one arm and allowed to explore the maze for 5 minutes. The sequence and number of arm entries were recorded. A spontaneous alternation was defined as consecutive entries into three different arms (e.g., A → B → C). The spontaneous alternation rate was calculated as follows: Spontaneous Alternation Rate = (Total number of arm entries−2)/Number of alternations×100%.

#### Elevated plus maze test

The elevated plus maze test was conducted to assess anxiety-like behavior in rats (29). The maze consisted of two open arms (50 cm long, 10 cm wide) and two closed arms (50 cm long, 10 cm wide, 40 cm high) arranged in a plus shape. The maze was elevated 1 meter above the floor. Each rat was placed in the center of the maze and allowed to explore for 5 minutes. The time spent in the open arms and the number of entries into the open arms were recorded using a video tracking system.

### Statistical analysis

Data were analyzed using GraphPad Prism (version 9.0). Escape latency and path length in the Morris water maze test were analyzed using two-way repeated-measures analysis of variance (ANOVA) followed by Bonferroni’s *post hoc* test. The time spent in the target quadrant, number of crossings over the former platform location, total distance traveled, number of line crossings, time spent in the center zone, number of rearing events, recognition index, spontaneous alternation rate, time spent in the open arms, and number of entries into the open arms were analyzed using one-way ANOVA followed by Tukey’s *post hoc* test. Data are presented as mean ± standard error of the mean. A p-value of less than 0.05 was considered statistically significant. Asterisks are used to denote statistical significance as follows: * indicates P < 0.05, ** indicates P < 0.01, *** indicates P < 0.001, and **** indicates P < 0.0001. The notation “ns” represents no statistical significance.

## Results

### Induction of periodontitis and *T. gondii* infection model

**Figure 1:**
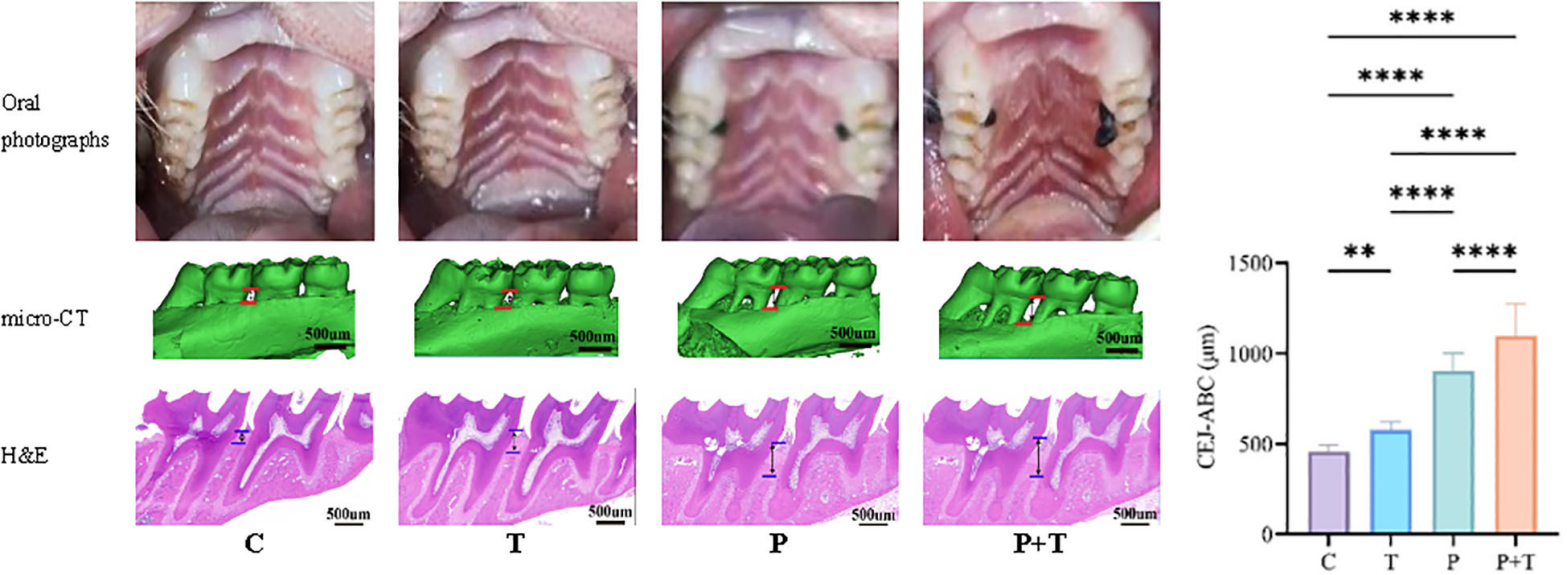
Multimodal Validation of Experimental Periodontitis, including oral photographs, micro-CT images, and H&E stained histological sections of the periodontal tissues. Micro-CT data show the distance from the cemento-enamel junction to the alveolar crest (CEJ–AC) measured at the distal root of the left maxillary first molar; n = 6 rats per group. Statistical differences were assessed by one-way ANOVA with Tukey’s *post-hoc* test. ** P < 0.01, **** P < 0.0001.

**Figure 2:**
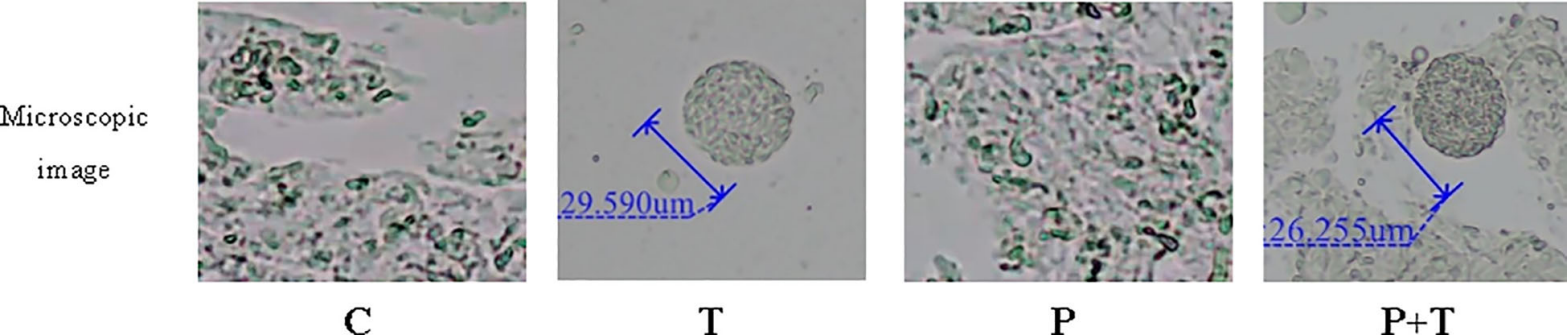
Confirmation of *T. gondii* Chronic Infection. Microscopic images of the brain tissue from rats in the T and P+T groups, had shown these representative *T. gondii* tissue cysts.

To ensure the validity of our experimental design, we first confirmed the successful induction of periodontitis and chronic *T. gondii* infection using multimodal assessments. Periodontitis was induced by combining silk ligature placement with repeated application of *P. gingivalis*. Oral examination revealed visible gingival inflammation, including redness, swelling, and bleeding on probing, in both the Periodontitis group and the Coinfection group, whereas the Control group and the *T. gondii* group exhibited healthy gingival tissue (**Figure 1**). Micro-CT quantification revealed significant alveolar bone loss at the distal root of the left maxillary first molar in both the Periodontitis group (902.89 ± 93.68 μm) and the Coinfection group (1098.53 ± 169.48 μm) compared with the Control group (456.43 ± 36.79 μm) (**Figure 1**). Histological analysis further confirmed periodontal pathology, showing dense inflammatory cell infiltration and disruption of periodontal ligament architecture in the Periodontitis group and the Coinfection group, and features absent in the Control group (**Figure 1**). Besides, chronic *T. gondii* infection was verified in the brain. Microscopic examination of brain sections from the *T. gondii* group and the Coinfection group revealed characteristic tissue cysts (**Figure 2**), which were absent in the Control group and the Periodontitis group. Together, these results confirmed the successful induction of both experimental periodontitis and chronic *T. gondii* infection in our coinfection model.

### Morris water maze test

The Morris water maze test was conducted to evaluate the spatial learning and memory abilities of rats in different experimental groups. Given that the platform was located in the fourth quadrant, a longer distance traveled and more time spent in this quadrant, as well as a higher number of crossings over the former platform location, are indicative of better spatial memory.

**Figure 3:**
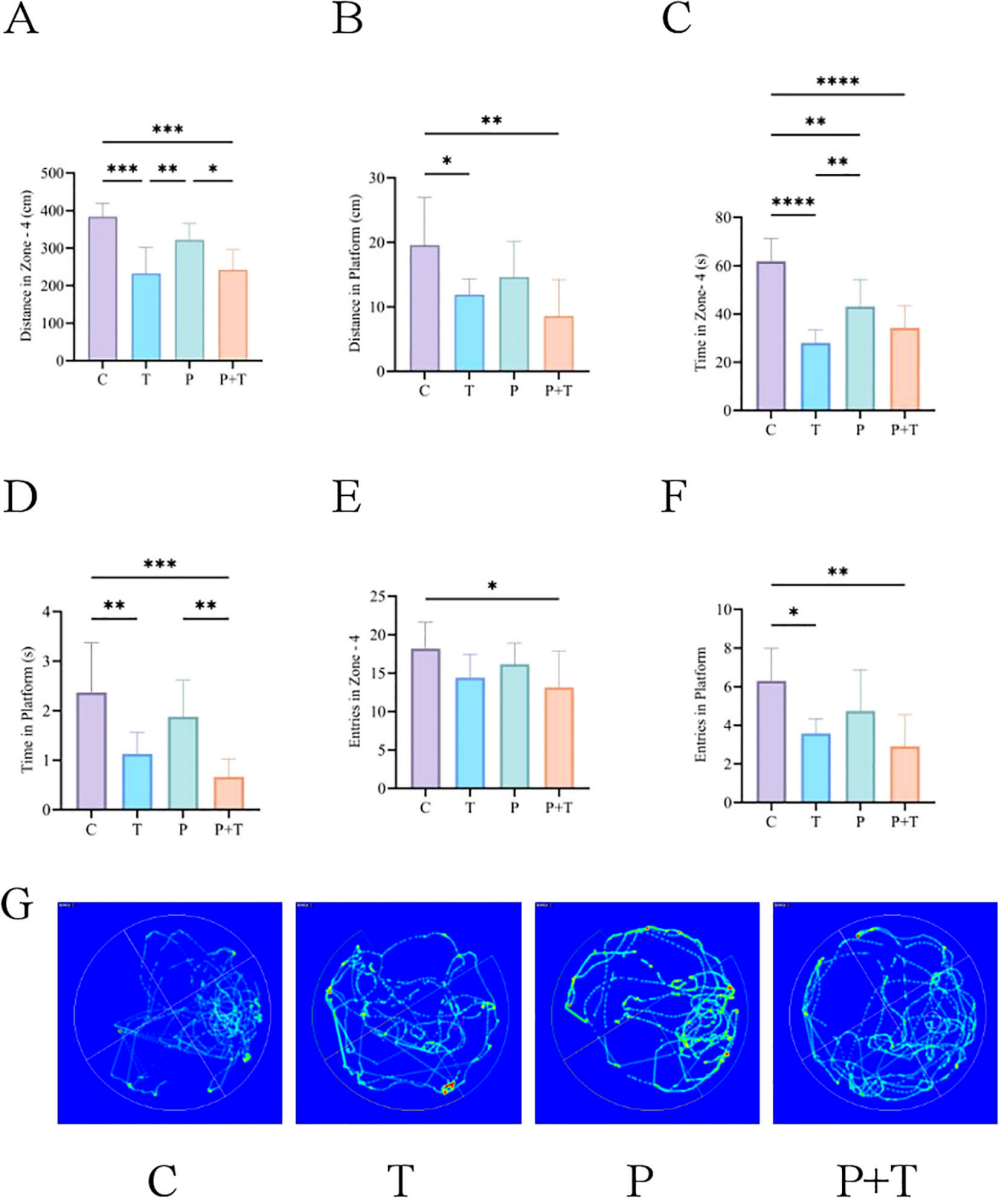
Results of the Water Maze Test. **(A)** Distance traveled in the target quadrant. **(B)** Distance traveled in the original platform area. **(C)** Time spent in the target quadrant. **(D)** Time spent in the original platform area. **(E)** Number of crossings into the target quadrant. **(F)** Number of crossings into the original platform area. **(G)** Water maze trajectories. * P < 0.05, ** P < 0.01, *** P < 0.001, **** P < 0.0001.

The results showed significant differences among the groups. Compared to the Control group, rats in the Periodontitis group and *T. gondii* group exhibited reduced distance traveled and time spent in the fourth quadrant (**Figures 3A–D**), as well as fewer crossings over the former platform location (**Figures 3E, F**), indicating impaired spatial learning and memory. The Coinfection group demonstrated the most severe deficits, with the shortest distance traveled, least time spent in the target quadrant, and fewest platform crossings. These findings suggest that *T. gondii* infection significantly impairs spatial learning and memory, with the effects being exacerbated in the presence of periodontitis.

The trajectory maps from the water maze test revealed that rats in the Control group and the Periodontitis group exhibited more focused movement patterns in the fourth quadrant and around the former platform location, indicating better spatial learning and memory abilities. In contrast, rats infected with *T. gondii* showed more dispersed movement trajectories, suggesting poorer spatial learning and memory capabilities (**Figure 3G**).

### Open field test

**Figure 4:**
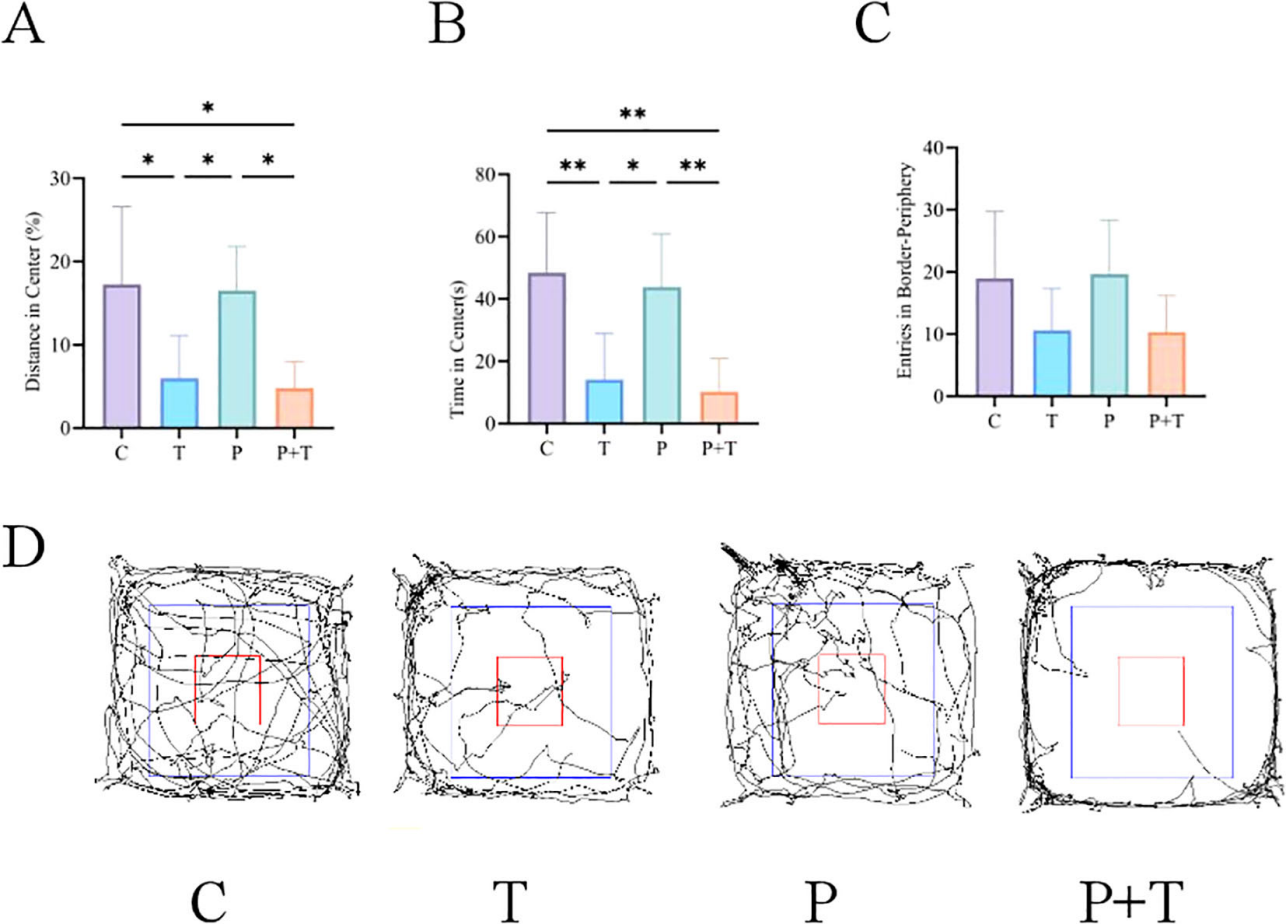
Results of the Open Field Test. **(A)** Total distance traveled. **(B)** Time spent in the center zone. **(C)** Number of line crossings. **(D)** Open field trajectories. * P < 0.05, ** P < 0.01.

The open field test was conducted to assess locomotor activity, exploratory behavior, and anxiety-like behavior in rats. The results are summarized as follows:

1. Total Distance Traveled: The total distance traveled by the rats reflects their overall locomotor activity. Animals that are more exploratory and less anxious tend to move more and cover a greater distance, while those in a depressive state often remain in the corners of the open field, resulting in shorter distances traveled. Compared to the Control group, rats in the *T. gondii* group exhibited significantly reduced total distance traveled, indicating decreased locomotor activity and potentially higher anxiety levels (**Figure 4A**). The Coinfection group showed the most pronounced reduction in total distance traveled, suggesting the most severe impact on locomotor activity. However, there was no significant difference between the Periodontitis group and the Control group in terms of total distance traveled.
2. Time Spent in the Center Zone: The time spent in the center zone versus the peripheral zone provides insights into the animal’s anxiety levels. Rats with higher anxiety levels tend to spend more time in the peripheral zone, while those with lower anxiety levels spend more time in the center zone. Compared to Control group, rats in the *T. gondii* group spent significantly less time in the center zone, indicating increased anxiety-like behavior (**Figure 4B**). The Coinfection group showed the most significant reduction in time spent in the center zone, suggesting the highest level of anxiety among the experimental groups. However, there was no significant difference between the Periodontitis group and the Control group in terms of time spent in the center zone.
3. Line Crossings: The number of line crossings, defined as the instances when all four paws of the rat enter an adjacent square, directly reflects the animal’s spontaneous activity and level of excitement. Reduced line crossings are often associated with depressive or anxious states. The *T. gondii* group had significantly fewer line crossings compared to the Control group, indicating lower levels of spontaneous activity (**Figures 4C, D**). The Coinfection group exhibited the fewest line crossings, further supporting the notion of heightened anxiety and reduced exploratory behavior. However, there was no significant difference between the Periodontitis group and the Control group in terms of line crossings.

These findings collectively suggest that *T. gondii* infection significantly increases anxiety-like behavior and reduces locomotor activity in rats, with the effects being more pronounced in the presence of periodontitis.

### Novel object recognition test

**Figure 5:**
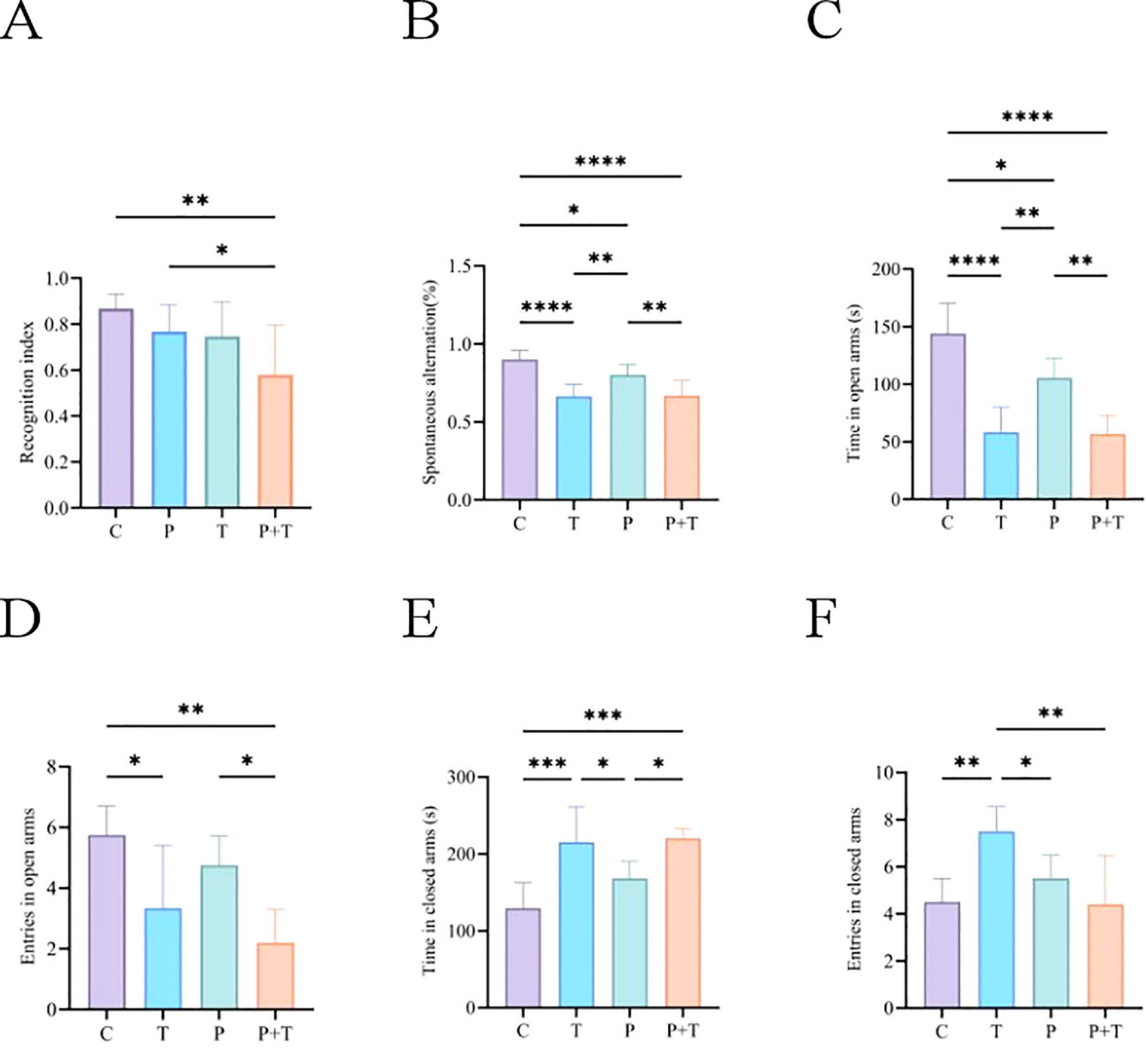
Results of the Novel Object Recognition Test. **(A)** Recognition Index. Results of the Y-Maze Test. **(B)** Spontaneous Alternation Rate. Results of the Elevated Plus Maze Test. **(C)** Time Spent in Open Arms. **(D)** Number of Open Arm Entries. **(E)** Time Spent in Closed Arms. **(F)** Number of Closed Arm Entries. * P < 0.05, ** P < 0.01, *** P < 0.001, **** P < 0.0001.

The novel object recognition test was conducted to evaluate cognitive function and memory in rats. A higher RI indicates better cognitive function and memory. The results showed the Coinfection group had significantly lower RIs compared to the Control group, indicating lower levels of cognitive function and memory (**Figure 5A**). However, there were no significant differences in RIs between the Periodontitis group and the Control group, nor between the *T. gondii* group and the Control group.

### Y-maze test

The Y-maze test was used to assess spatial working memory in rats. A higher spontaneous alternation rate indicates better spatial working memory. The results showed the *T. gondii* group, Periodontitis group and Coinfection group had lower spontaneous alternation rates compared to the control group, indicating impaired spatial working memory (**Figure 5B**).

### Elevated plus maze test

The elevated plus maze test was conducted to assess anxiety-like behavior in rats. Anxiety-like behavior was indicated by reduced time spent in the open arms and fewer entries into the open arms. The results showed compared to the Control group, the Periodontitis group spent more time in the open arms, and both the *T. gondii* group and the Coinfection group had time less time spent in the open arms and fewer entries into the open arms, indicating anxiety-like behavior (**Figures 5C–F**).

## Discussion

The present study show that periodontitis amplifies *T. gondii*–induced cognitive function damage and exacerbates spatial-memory deficits and anxiety-like behavior in rats. These findings suggest that periodontal status modulates the central-nervous-system impact of chronic *T. gondii* infection; they do not establish causality for periodontitis alone. Our findings align with previous studies demonstrating the impact of *T. gondii* infection on behavioral and cognitive outcomes, further supporting the hypothesis that this parasite can influence CNS function (31–33).

One of the primary mechanisms through which *T. gondii* infection may impair cognitive function is via the induction of neuroinflammation. While previous studies have shown that *T. gondii* infection triggers the release of pro-inflammatory cytokines (IL-1β, IL-6, TNF-α) that can cross the BBB and activate microglia (34, 35), direct evidence of neuroinflammation (e.g., microglial activation, neuronal damage markers) was not obtained in the current study. Thus, neuroinflammation remains a plausible but not fully demonstrated pathway, and future work should include hippocampal cytokine profiling and microglial histomorphometry to substantiate this mechanism. In our study, the significant impairment in spatial learning and memory observed in *T. gondii*-infected rats is likely due to neuroinflammation induced by the parasite.

The choice of inoculation routes was based on the natural infection niches of each pathogen. *P. gingivalis* is an obligate oral surface bacterium, and gingival sulcus inoculation with ligature placement recreates the subgingival biofilm environment that initiates human periodontitis (26). Besides, *T. gondii* tissue cysts are ingested orally in nature, so oral gavage reliably establishes chronic CNS infection, mimicking human exposure (27). *T. gondii* infection can also disrupt the integrity of the BBB, facilitating the entry of peripheral immune cells and inflammatory mediators into the CNS. This disruption is further exacerbated in the presence of periodontitis, which itself induces systemic inflammation and can compromise the BBB (36). The combined effect of *T. gondii* infection and periodontitis likely contributes to the more pronounced cognitive deficits observed in the Coinfection group.

*T. gondii* infection has been shown to modulate neurotransmitter levels in the brain, particularly dopamine and glutamate (12). Elevated dopamine levels have been reported in *T. gondii*-infected mice, which may contribute to the behavioral changes observed in these animals (31). Additionally, alterations in glutamate signaling can disrupt synaptic plasticity and long-term potentiation, critical processes for learning and memory. Our findings of impaired cognitive function in *T. gondii*-infected rats suggest that neurotransmitter modulation may play a significant role in the observed behavioral deficits (1).

The induction of anxiety-like behavior in *T. gondii*-infected rats is consistent with previous studies linking this parasite to increased anxiety and depression-like behaviors (32). The underlying mechanisms may involve neuroinflammation and neurotransmitter modulation, both of which can influence mood and anxiety-related behaviors. The results of elevated plus maze and open field tests in our study indicate that *T. gondii* infection significantly increases anxiety-like behavior in rats.

Periodontitis, characterized by chronic inflammation of the oral tissues, has been implicated in various systemic diseases, including the cognitive decline and the neurodegenerative disorders (17). The presence of periodontitis in our study exacerbated the cognitive and behavioral deficits induced by *T. gondii* infection. This interaction may be mediated through several pathways. Firstly, periodontitis induces systemic inflammation, which can exacerbate the neuroinflammatory response triggered by *T. gondii* infection. The combined inflammatory burden may lead to more pronounced disruption of the BBB and increased infiltration of immune cells into the CNS (14). Secondly, periodontitis is associated with a dysbiotic oral microbiome, which can influence systemic health through the release of bacterial virulence factors and metabolic byproducts (16). These microbial products can further contribute to systemic inflammation and neuroinflammation, exacerbating the effects of *T. gondii* infection.

Our findings have important implications for understanding the pathophysiology of neuropsychiatric disorders. The association between *T. gondii* infection and conditions such as schizophrenia and bipolar disorder has been widely documented. The exacerbation of cognitive and behavioral deficits in the presence of periodontitis suggests that oral health may play a significant role in modulating the impact of *T. gondii* infection on CNS function (18, 19). This highlights the importance of maintaining good oral hygiene as a potential preventive measure against cognitive decline and neuropsychiatric disorders.

Notably, there is no published literature directly reporting a synergistic interaction between *T. gondii* and *P. gingivalis*, making our study exploratory in nature. The observed exacerbation of cognitive and behavioral deficits in the Coinfection group requires validation through independent studies with larger sample sizes to enhance statistical power. Additionally, while we demonstrated confirmed periodontitis and *T. gondii* infection, we did not isolate the specific contributions of stress, sleep, nutritional status, or gut dysbiosis—factors that may interact with systemic and neuroinflammation to influence behavior. While our study provides valuable insights into the effects of *T. gondii* infection and periodontitis on cognitive function and behavior, several areas warrant further investigation. First, future studies should focus on elucidating the specific molecular mechanisms underlying the observed behavioral and cognitive deficits. This includes investigating the role of neuroinflammation, neurotransmitter modulation, and BBB disruption in more detail. Second, the findings from animal models need to be translated to human studies to confirm the association between *T. gondii* infection, periodontitis, and neuropsychiatric disorders. Large-scale epidemiological studies and clinical trials are needed to establish causality and explore potential therapeutic interventions. Third, longitudinal studies are needed to assess the long-term impact of *T. gondii* infection and periodontitis on cognitive function and behavior. This will facilitate the understanding of progression of these effects over time and identifying critical periods for intervention.

An important limitation of this study is that we did not directly assess the presence of *P. gingivalis* or its virulence factors within the brain. As suggested by previous studies, periodontal pathogens like *P. gingivalis* can translocate to the brain, potentially contributing to neuroinflammation and neurodegeneration (36–43). A compelling question for future research is whether such central nervous system invasion by *P. gingivalis* occurs in our coinfection model. Future studies employing techniques such as immunohistochemistry with specific antibodies on brain tissue will be essential to investigate this potential mechanism directly.

## Conclusions

Our study demonstrates that *T. gondii* infection significantly impairs cognitive function and induces anxiety-like behavior in rats, effects that are exacerbated in the presence of periodontitis. These findings highlight the potential role of the periodontitis in mediating the impact of *T. gondii* infection on CNS function. Importantly, the study design does not establish causality for periodontitis alone, as behavioral changes were most pronounced in the context of co-infection. Maintaining good oral health may serve as a potential preventive strategy to mitigate the cognitive and behavioral consequences of *T. gondii* infection. Future research should focus on elucidating the underlying molecular mechanisms using larger cohorts and integrated neuroimmunological profiling, and translating these animal findings to human epidemiological and clinical studies.

## Data Availability

The raw data supporting the conclusions of this article will be made available by the authors, without undue reservation.
